# An Intervention to Promote Medication Understanding and Use Self-Efficacy: Design of Video Narratives for Aging Patients at Risk of Recurrent Stroke

**DOI:** 10.2196/11539

**Published:** 2019-03-21

**Authors:** Jamuna Rani Appalasamy, Joyce Pauline Joseph, Siva Seeta Ramaiah, Kia Fatt Quek, Anuar Zaini Md Zain, Kyi Kyi Tha

**Affiliations:** 1 Jeffrey Cheah School of Medicine and Health Sciences Monash University Malaysia Bandar Sunway, Selangor Malaysia; 2 Department of Neurology Hospital Kuala Lumpur Ministry of Health Kuala Lumpur Malaysia; 3 Medical Department Subang Jaya Medical Center Sunway Malaysia

**Keywords:** Delphi technique, self-efficacy, stroke, personal narratives, video-audio media, beliefs

## Abstract

**Background:**

The debilitating effects of recurrent stroke among aging patients have urged researchers to explore medication adherence among these patients. Video narratives built upon Health Belief Model (HBM) constructs have displayed potential impact on medication adherence, adding an advantage to patient education efforts. However, its effect on medication understanding and use self-efficacy have not been tested.

**Objective:**

The researchers believed that culturally sensitive video narratives, which catered to a specific niche, would reveal a personalized impact on medication adherence. Therefore, this study aimed to develop and validate video narratives for this purpose.

**Methods:**

This study adapted the Delphi method to develop a consensus on the video scripts’ contents based on learning outcomes and HBM constructs. The panel of experts comprised 8 members representing professional stroke disease experts and experienced poststroke patients in Malaysia. The Delphi method involved 3 rounds of discussions. Once the consensus among members was achieved, the researchers drafted the initial scripts in English, which were then back translated to the Malay language. A total of 10 bilingual patients, within the study’s inclusion criteria, screened the scripts for comprehension. Subsequently, a neurologist and poststroke patient narrated the scripts in both languages as they were filmed, to add to the realism of the narratives. Then, the video narratives underwent a few cycles of editing after some feedback on video engagement by the bilingual patients. Few statistical analyses were applied to confirm the validity and reliability of the video narratives.

**Results:**

Initially, the researchers proposed 8 learning outcomes and 9 questions based on HBM constructs for the video scripts’ content. However, following Delphi rounds 1 to 3, a few statements were omitted and rephrased. The Kendall coefficient of concordance, W, was about 0.7 (*P*<.001) for both learning outcomes and questions which indicated good agreement between members. Each statement’s Cronbach alpha was above .8 with SD values within a range below 1.5 that confirmed satisfactory content and construct validity. Approximately 75% (6/8) of members agreed that all chosen statements were relevant and suitable for video script content development. Similarly, more than 80% (8/10) of patients scored video engagement above average, intraclass correlation coefficient was above 0.7, whereas its Kendall W was about 0.7 with significance (*P*<.001), which indicated average agreement that the video narratives perceived realism.

**Conclusions:**

The Delphi method was proven to be helpful in conducting discussions systematically and providing precise content for the development of video narratives, whereas the Video Engagement Scale was an appropriate measurement of video realism and emotions, which the researchers believed could positively impact medication understanding and use self-efficacy among patients with stroke. A feasibility and acceptability study in an actual stroke care center is needed.

**Trial Registration:**

Australian New Zealand Clinical Trials Registry ACTRN12618000174280; https://www.anzctr.org.au /Trial/Registration/TrialReview.aspx?id=373554&isReview=true

## Introduction

### Background

Medication nonadherence is prevalent at large especially in major chronic diseases, despite patient education and advanced knowledge and methods [1]. Regardless of a definite health economic impact, current endeavors of patient education interventions still appear to be inadequate [2]. Globally, stroke prevalence is not exempted from this cliché of medication nonadherence [3]. A similar situation and increasing aging population of poststroke patients in Asian countries, such as Malaysia, urge for robust and cost-effective patient education measures [4,5]. So far, insufficient patient education intervention reported the effects of video education in patients with chronic medical conditions such as stroke. It is also unknown if personal stories related to stroke medication management can enhance self-efficacy and promote adherence to stroke preventative medication or control stroke risk factors.

In educational strategy, 75% of information is engaged visually and about 13% of it is engaged using our hearing senses [6]. Hence, when a patient sees and hears a video, they have a higher probability of comprehending and reflecting the information. Videos delivered via television format allows viewers of any age group to grasp information at a continuous pace or in a relaxed and inducive environment [6].

The researchers proposed a patient education intervention at an outpatient stroke clinic as it may be a perfect venue for focused recurrent stroke education because it provides access to a common variation of people who are at high risk for recurrent stroke. In a quest for cost-effectiveness, the researchers utilized the prolonged waiting time in the clinic as an opportunity to deliver the intervention adjunct to the current medication therapy adherence clinic’s (MTAC) effort that may benefit the patients with stroke. Time spent in the waiting area is a potential period for patients to gain knowledge and confidence in managing their medication [7]. This educational approach may be valuable to patients who were not inclined to electronic communication devices, lacking internet facility, or to those who depended on an external motivational environment such as peers.

The researchers believed that video narratives shown simultaneously with patient education modules are expected to have a positive impact on self-efficacy. Consequently, if the video is incorporated with theoretical behavioral constructs, it could induce self-reflection and simulation by a role model. In addition, if the video is repetitively seen, a *persuasive power* would be instilled whereby the individual’s perception influenced by previous learning experience would have a change. The Social Learning Theory explains that an individual’s behavior depends on the conditioning of the mind, influenced by his or her environment, which then controls the action of the doer [8]. The planned environment here was the video viewing activity in the waiting area of an outpatient stroke clinic. Besides, the role model impact would be more significant if the actual people who experienced the events delivered the video narratives [9]. It makes the content’s objectives realizable and might induce the patient’s confidence in justifying what was said, seen, or heard in the video.

### Objectives

This study hypothesized that providing video narratives incorporated with theoretical behavioral constructs adjunct to the existing MTAC’s patient education effort, informational brochures, counseling, and medication review would result in better stroke awareness, medication understanding, and use self-efficacy toward improved adherence. This study was the intervention development and validation phase of a randomized controlled trial (universal trial number: U1111-1201-3955) [10]. This study described the processes involved in the video narrative’s development and validation.

## Methods

### The Delphi Method

The Delphi method originated from RAND Corporation studies from the 1950s and aimed to develop a reliable technique to obtain consensus from experts. Since then, many researchers have applied this organized method for expert problem-solving issues. They have also developed systematic guidelines of the process and analysis of the Delphi Method [11,12].

The researchers in this study applied a Delphi method to obtain anonymous consensus on learning outcomes, Health Belief Model (HBM) constructs, and content of video scripts which took place from October 2017 to December 2017 among experts experienced in stroke patient education. The consensus procedure incorporated 3 rounds of questionnaires via email to finalize expert panelists’ viewpoints.

The process started with literature findings on the local need for stroke survivors. Most patients’ crucial need encompassed feelings of being independent to have a good quality of life, reducing the severity and preventing recurrent stroke [13-16]. To be able to achieve these aims, the patient would require utmost confidence and self-efficacy. Moreover, the learning objectives must be able to reflect similar insights and align with the objectives of patient education of recurrent stroke preventative treatment and management guideline of Malaysia [14,15].

Fundamentally, the design of the content was based on the most widely used framework, HBM [17]. HBM has outlined few health behavior constructs that guide a patient’s decision making ability such as perception of the risk of contracting the illness and how an adverse effect of illness affects their life, balancing the pros and cons of the actions if taken and prompts for the action. These HBM conditions led the researchers to develop an ideal set of questions as learning objectives to develop the video scripts. Other than scripts, presenting it as a video format was a valuable prompt for the patients with stroke *to take action* on their medication-taking habit.

The core of the Delphi method was the selection of a knowledgeable and experienced expert panel of members within the specific need of content development [18]. Therefore, the researchers invited members of the stroke community and health care professionals who then gave consent via email after provision of information and a brief explanation by the researchers.

The expert panel team of 8 comprised 2 neurologists, 2 pharmacists, 2 medical educationists, and 2 patients who had experienced a stroke. The neurologists were selected based on their 10 to 12 years of professional experience of diagnosing and prescribing medications to patients with stroke. The pharmacists were also selected based on their 10 to 12 years of professional experience of reviewing and dispensing prescribed medications to patients with stroke at the hospital and community level. Whereas, the medical educationists, who were also knowledgeable in developing curricular pedagogy, contributed to the suitability of learning outcomes for stroke according to local context and sensitivity. Finally, the patients with stroke for about 5 years had experiences and an awareness of the need for emotional support to enhance self-efficacy.

There is no specific sample size recommendation for the Delphi method in this area of study as different disciplines and purpose of discussion often result in dissimilar response rates and time [19,20]. However, the researchers ensured all members were homogenous of a specific niche for content development [18] as each of them were bilingual, had relevant knowledge about stroke, were well-versed in stroke preventative management and actively involved with the latest stroke research update and stroke community undertakings, and were willing to volunteer to respond to up to 3 rounds of discussions.

### The Development of the Video Narrative Scripts

A fruitful discussion with the panel of experts led to the video narrative script development. The researchers developed the scripts in English and translated them into the Malay language with the help of a professional bilingual translator. Then, back translation was performed by another bilingual researcher who was not exposed to the initial scripts to verify the similarity of meanings. Both scripts (a neurologist’s and a patient’s version) addressed a brief summary of (1) the debilitating impact of stroke; (2) related risk factors of recurrent stroke, its prevention strategy, and benefit; (3) belief in self-confidence; and (4) real-life cues of successful recovery regardless of the severity of stroke. The Flesch-Kincaid reading level for the narrative scripts scored an average grade level of 6 [21]. Though each script was short (planned to be narrated within 2 min), it was precise with motivational aspects according to the behavioral constructs and was presented as a self-reflection story.

### The Development of the Video Narratives

The researchers believed that it was ideal and realistic to have actual actors (ie, neurologist and a patient who had experienced a stroke) to narrate the scripts. Meanwhile, the video was taken at the Arts and Social Sciences School, Monash University, Malaysia, with the help of a technical officer. They narrated each video script, both in English and Malay language within 2 min, and the manner of speech was according to communication principles [22]. The narration and video footage were at a sensible pace with several pauses and facial expressions showcasing appropriate emotion. The researchers also highlighted the videos with written captions and subtitles with a readability level of 6 [21]. A freelance video designer edited the videos using Movavi Video Editor 14 (Version 10.0.0; Obscure Reference Generator (Version 2.1; Shareware, 2014). The videos were repeatedly edited after several rounds of comments on visuals, sound clarity, and presentation style.

### Data Collection and Analysis

#### Delphi Method: Round 1

The researchers drafted the initial narrative script content guide from literature findings, which comprised 8 learning outcomes and 9 HBM-related questions linked to individual perceptions, cues to action, the likelihood of action, and self-efficacy. The panel of experts was given options (ie, yes: to agree to accept or no: do not agree to accept) and an open-ended question to add any other relevant information to the list or justify any redundancy. Hence, this round helped to establish the initial content and construct development of the list, clarification of meaning, and rephrasing or merging of a redundant statement. They were given 3 weeks’ time to respond to the Delphi method coordinator.

It was accepted that, approximately, an 80% agreement from the panel (ie, 6 or 7 out of 8 experts) for response frequencies for each learning outcome and HBM question was to be accepted or omitted. This percentage cut off was an appropriate reference point to attain content and construct validity [23]. Hence, the researchers removed those statements that were not meeting about 80% agreement, whereas the rest of the statements and HBM construct questions were modified, rephrased, or merged based on the experts’ feedback. Then the list was reedited in a survey questionnaire format and was emailed to the experts for Delphi method round 2.

#### Delphi Method: Round 2

The researchers repeated the same procedures and timeline as the previous discussion except that the panel of experts was asked to rank the level of relevance using a 7-point Likert scale (ie, 1: not at all relevant and 7: extremely relevant). They were asked to justify their choice of rank if it was 4 points and lower. Kendall W coefficient of concordance was used to measure the nonparametric rankings [24] for a better affirmation of content and construct validity. According to Kendall, the W value ranges from 0 to 1 (ie, 0: no consensus and 1: full consensus) with 0.7 and greater indicating strong agreement so that specific weaker agreement could be scrutinized and relooked to avoid bias and force agreement. Besides, an SD of below 1.5 was also considered to add value to the consensus compared with a percentile of agreement [25]. *P* values less than .05 are considered statistically significant.

The coordinator received comments and feedback to rephrase a few statements to illustrate appropriate meanings. The coordinator asked the expert panelists if they were willing to continue the rounds until the W value rises and all agreed. Hence, a final edition of learning outcomes and HBM questions were resent via an online survey questionnaire for the Delphi method round 3 discussion.

#### Delphi Method: Round 3

Round 2 discussion and analysis produced a summary of responses and clarification from the panel of experts, which gave an overall picture of final scoring and the current level of consensus of the *weaker strength* statements. The coordinator decided to run the final round of discussion, round 3, and the purpose was to hint the panel experts to confirm and justify revision of specific individual scores, which showed some discrepancies. Inter-rater reliability was determined with the ICC, whereby a 2-way mixed model (fixed raters) with absolute agreement was applied. An ICC value with 0.7 and greater indicated moderate to good reliability. W and SD values were then calculated, and subsequently, a full-detailed report of the discussion was sent to all expert members.

### The Validity and Reliability of Video Narrative Scripts and Videos

A purposeful sample of 10 bilingual patients with stroke (within the inclusion and exclusion criteria of the trial) were requested to provide written feedback on the comprehension of the English and Malay video narratives scripts. The informed and consented patients were asked to reply either via email or via a prepaid postal service. Their responses contributed to face and content validity. They also viewed the video narratives in both languages and responded to the Video Engagement Scale (VES) that was presented to them face-to-face during their follow-up clinic visit. Test-retest was not appropriate as these patients were exposed to patient education materials, which could affect their follow-up responses. We expected occurrences of revision in every round of iteration. Therefore test-retest was not applicable to the Delphi method.

To the researchers’ knowledge, there were no fixed guidelines to validate a video narrative for patient education; however, there has been a link between the construct of engagement and persuasive communication [26]. Therefore, the researchers adopted the VES to obtain feedback on the ecological validity of the video narratives [27]. The VES has been validated with right internal consistency, test-retest reliability, and content validity, and the authors suggested to use it to measure ecological validity and external validity of video vignettes [27]. VES was also developed based on videos with multiple cases and shots; therefore, this scale would be suitable to be related to emotion and motivation. The patients’ ratings contributed to the ICC and Kendall W value, whereas Cronbach alpha above .7 indicated the accepted internal consistency of response ratings. All statistical analyses were done using IBM SPSS software version 22). Data preprocessing was done to maintain data quality such as normalization and double data entry to prevent errors, missing values, or inconsistent codes.

### Ethics Approval

Approvals for this development and validation study have been obtained from the Malaysian Medical Research and Ethics Committee (NMRR ID-15-851-24737) and the Monash University Human Research Ethics Committee (ID 9640) whereas the MyStrokeStory trial was registered with the Australian New Zealand Clinical Trials Registry (ACTRN12618000174280; universal trial number U1111-1201-3955).

## Results

### The Delphi Method

The researchers made no addition to the initial draft of the learning outcomes and HBM questions before the Delphi method round 1. We omitted statements that were redundant, had less than 80% agreement (ie, What is a stroke? How serious is having a stroke?), or were rephrased (ie, How common is a stroke? to Who is at high risk of stroke?). Whereas, few other statements or questions had only a minor correction. Therefore, 8 learning outcomes and 9 HBM questions were edited to 6 statements with 6 questions each for the Delphi method round 2.

In round 2, the W value was below 0.7. The mean ranking for learning outcomes and HBM questions also varied (ie, 2 experts were asked to justify their low score for learning outcomes and HBM construct questions 1 and 2).

However, in round 3, the list of learning outcomes and HBM questions was finalized (Table 1). Kendall coefficient of concordance, W, of approximately 0.7 indicated a firm agreement, and SD values below 1.5 confirmed satisfactory content and construct validity of learning outcomes and HBM questions. However, a reliability test was computed independently for round 3, whereby Cronbach alpha was above .7, which indicated good internal consistency; items on the finalized learning outcomes and HBM construct questions were developed on the similar idea or construct (Table 2).

### The Validity and Reliability of Video Narrative Scripts and Videos

The researchers received positive feedback on the scripts (ie, good script, short and meaningful, and direct points), but there were not many comments on the structure of sentences or usage of words. Therefore, the researchers concluded that the scripts were suitable to the local context; hence, the narrative scripts were finally confirmed.

**Table 1:** The finalized video narrative scripts’ learning outcomes and questions parallel with the Health Belief Model constructs.

Health Belief Model constructs	Learning outcomes	Questions
Individual perception: Perceived susceptibility; Perceived severity	1. To be able to recognize and understand stroke cause, symptoms, and effects 2. To understand the burden of stroke	1. What happens to you when you have a stroke? 2. Who is at high risk of stroke?
Likelihood of action: Perceived benefit; Perceived barrier	3. To understand lifestyle risk factors of stroke 4. To acquire information in medication understanding and use	3. How do you prevent another stroke? 4. How do medications reduce the risk of another stroke?
Self-efficacy	5. To understand and acquire skills of medication understanding and use self-efficacy after a stroke	5. How do you ensure your medication works for you?

**Table 2:** Final analysis of the Delphi method (n=8).

Raters	10 items, mean^a,b,c^
Member 1	4.4
Member 2	6.2
Member 3	4.6
Member 4	4.4
Member 5	5.5
Member 6	3.4
Member 7	5.4
Member 8	5.4

^a^Cronbach alpha: .908.

^b^Intraclass correlation coefficient (95% CI): 0.733 (0.384-0.919).

^c^*P*<.001.

**Table 3:** The Video Engagement Scale scores (n=10).

Raters	15 items, mean^a,b,c^
Patient 1	5.3333
Patient 2	5.6000
Patient 3	5.7333
Patient 4	5.9333
Patient 5	6.4000
Patient 6	6.5333
Patient 7	6.7333
Patient 8	6.8667
Patient 9	6.8667
Patient 10	6.6667

^a^Cronbach alpha: .925.

^b^Intraclass correlation coefficient (95% CI): 0.797 (0.572-0.921).

^c^*P*<.001.

The VES scores were above average, which exhibited a good link with perceived realism (Table 3). Out of 10 patients, more than 80% of them agreed on the validity of emotional and motivational aspects of the video narratives with a Kendall W value of 0.63 and SD average below 1.5. However, the Cronbach alpha above .7 indicated satisfactory reliability for all videos, which indicated good internal consistency; the emotional and motivational levels were on a similar agreement.

## Discussion

### Principal Findings

This study explicitly developed and validated video narratives to be used as intervention materials in a randomized controlled trial [10] whereby the researchers would be able to monitor the effect of narration from a doctor and patient with stroke on medication understanding and use self-efficacy of patients who have experienced stroke. The scripts were a general reflection of recurrent stroke and its underlying comorbidity management with a mix of motivation and advice, which hoped to trigger a sense of self-efficacy among patients with stroke to understand and use prescribed medication. The video narratives underwent rigorous processes (ie, development of script guidelines as in learning outcomes and HBM questions, bilingual script development, and video editing) and few phases of satisfactory validation: face validity, content and construct validity (Delphi method), reliability test, and ecological validity (video engagement with bilingual patients). Hence, these video narratives were considered valid and reliable to be presented to patients with stroke with a projected aim to avert stroke risk factors and, in the longer term, prevent recurrent stroke. Videos with patient narratives have the persuasive strength of behavior modification especially if culturally sensitive and embedded with a role model effect. Professional actors, good script constructs and content, appropriate language, and video presentation style play a part in delivering an impactful source in a behavioral intervention [28-30].

### Strength and Limitations

There were some apparent limitations in this video narrative development. Face-to-face discussion was unable to be carried out in the Delphi method rounds owing to the lack of interval time and slow responses from the expert panel despite constant reminders. Hence, the Delphi method discussion ended in round 3 whereby force agreement would have occurred. The researchers were also aware that face validity and video engagement responses lacked the required number of participation from poststroke patients because of specific inclusion and exclusion criteria via purposive sampling method. Therefore, the video narratives’ validation and study aim were skewed toward particular samples only, and hence, results could not be generalized to the whole population of patients with stroke. In addition, responses from nonbilingual patients were also not assessed owing to the delay during the purposive sampling period and having the VES available in the English version only.

Nevertheless, the Delphi method proved to be a versatile and helpful technique in conducting discussions systematically and reaching a consensus unanimously, eliciting precise ideas, and providing rich, in-depth data in defining an intervention strategy. In addition, the video narrative development processes were found to be useful as a guideline for other behavioral studies, which use video as their intervention, samples with chronic illness, and study sites other than health care centers.

The researchers believed that *no stone had been left unturned* in this development and validation process. The VES had helped to reveal the preliminary understanding of the patients’ video engagement styles and emotions that were being affected (ie, realism, empathy, and awareness); however, we believed that bigger samples would produce far more significant data. The researchers recommend that the VES be summarized and translated in various languages in the future to test its effectiveness in distinguishing the video engagement style of multicultures. A future test of the video narratives’ feasibility and acceptability in an actual stroke care center would undoubtedly add significance to its validation and effectiveness.

## Abbreviations

HBM: Health Belief Model
ICC: intraclass correlation coefficient
MTAC: medication therapy adherence clinic
VES: Video Engagement Scale
